# Unqualified Assistants and Death Certificates

**Published:** 1889-10-05

**Authors:** 


### Unqualified Assistants and Death Certificates.

By Index.

In The Hospital of the 7th ult. there is a short paragraph among the "Gleanings" to the effect that the Roman Catholic priest of Chasetown had accused Dr. Gettings, poor law medical officer of one of the Lichfield districts, of allowing his unqualified assistant to attend his (parish) patients, and of giving unsatisfactory death certificates; that Dr. Gettings had given explanations to the entire satisfaction of the local guardians, and stated that the accusations had been prompted by personal malice. As two important principles are involved here, and in which I take much interest, I wrote these two gentlemen respectively to favour me with the plain facts of the case, not making much of any local colouring that may have been given to it. Dr. Gettings has declined to give me any information until he knew whether I am "a hostile or friendly critic," but from the Rev. F. MacCarrick I have got published copies of the "explanations" of the former and of the latter's "reply," sent to the Local Government Board. Having read these carefully, I beg to ask the insertion of my remarks thereon in The Hospital for the sake of the points at issue. Dr. G. admits the chief facts urged against him by Mr. MacCarrick that his unqualified assistant did attend his parish cases (under his own direct and personal supervision), and that he signed certificates of death filled in with the dates of attendance of his assistant, calling this a "possibly technical error." It would have been a very exceptional instance indeed if the parish poor were not relegated very generally to the care of the assistant, unqualified or otherwise; and this practice of signing death certificates on attendance thus given is, I might say, universal. Now, is either of these things legal or just? That many (legally) unqualified assistants are good men, well up in the practical knowledge and routine of their profession, cannot be doubted, though not versed in the teachings of bacteriology or microscopic pathology, but that too many more are utterly and hopelessly strangers to these things, and in no ways careful to learn them, must also be admitted. Too frequently are they the tools of their employers, of any meanness, trickery, or unprofessional dodge whereby another medical man may be injured and his own interests promoted. The shabby, unbecoming work is done by the miserably abject assistant, who takes the odium, while his employer, with all the indignant protestation (if need be) of innocence, pockets the results.

Dickens, who sketches out the life of the poor lawyer's clerk, could do justice to the doctor's unqualified assistant, toiling, drudging on, and submitting to the every behest of his employer. Nor do I confine these statements to the unqualified man, for his more advanced and legally lettered compeer frequently allows himself to be like used. How have we the profession degraded in the eyes of the public and parish districts taken by the medical man at miserably low salaries, because the attendance is not to be given, except in most unfrequent instances, by themselves, but by assistants paid and treated accordingly. Whether such attendance be *legal* is a point to be determined by the Local Government Board, but whether the local guardians do well in sanctioning services which they would in their own sickness very generally peremptorily refuse (if indeed it were in a thoughtless moment offered them by the principals) may very reasonably be doubted. For the case of club and private patients, there may be no discussion, because these have themselves the power to reject the attendance so proffered, but who ever heard of a pauper insisting upon the (presumably) more efficient attendance of the doctor himself? Guardians feel themselves bound to inspect and, if need be, refuse the goods of the grocer or other tradesman if not up to estimate, but the medical care of their sick poor is quite well enough done (so they seem to think) by the inferior service of their paid officeres assistant.

As to the legality of requiring death certificates on the attendance of unqualified assistants, there may be less doubt. If such be not legally qualified to practice, his attendance cannot be legally certified. The maxim, *agit per aliquid agit per se*, holds good in ethics, but the Births and Deaths Registration Act stipulates in its wording of the certificate that the attendance shall have been by the party signing. It may be at once admitted that here Dr. Gettings has acted only according to what may be called almost unexceptional practice, but should such practices be allowed? Doubtless the fact of a death certificate being signed only on the attendance of a legally qualified man would necessitate a decided increase in the salaries given to these medical officers, and the sooner this is done the better. If *cheap* services be, proverbially, always the dearest, such increased salaries would be an ultimate saving to the poor rates, and, if not, what is the worth of that increased ability to treat disease which legal qualifications are held to indicate?

I would ask just space to say that though thus delivering myself, I have more than once refused to take a part towards preventing the practice of unqualified men. The evil has been allowed to grow, and to tear it thus violently (as it were) up by the root would cause much suffering to many. But, by all means, let a beginning be made by some well-considered scheme to do away with a system (I am now referring specially to our poor law service) which is not only wrong in itself, but the cause of much wrong to others, and among these, not certainly the least suffering, are our helpless poor.

